# THE COMPARISON OF DUAL TASK TIME AMONG APOE CARRIERS AGAINST NON-APOE CARRIERS IN AGING ADULTS

**DOI:** 10.1093/geroni/igac059.2790

**Published:** 2022-12-20

**Authors:** Cody Diehl, Anthony Campitelli, Megan Jones, Joshua Gills, Sally Paulson, Jordan Glenn, Kelsey Bryk, Michelle Gray

**Affiliations:** University of Arkansas, Fayetteville, Arkansas, United States; University of Arkansas, Fayetteville, Arkansas, United States; University of Arkansas, Fayetteville, Arkansas, United States; Rutgers University-Newark, Jersey City, New Jersey, United States; St. Elizabeth Healthcare, Cincinnati, Ohio, United States; Neurotrack Technologies, Redwood City, California, United States; Neurotrack Technologies, Inc., Redwoods City, California, United States; University of Arkansas, Fayetteville, Arkansas, United States

## Abstract

No single variable has been identified as the sole factor for predicting an individuals’ declining health status. In order to predict mental or physical decline in older adults, researchers have designed tasks that include both a physical and mental or cognitive demand known as dual task (DT). The purpose of implementing dual task is to assess the possible changes in accuracy when a second task gets compounded with a given single task. Our study consists of males and females aged from 45 to 75 years who have risk factors for dementia. Each subject was randomly assigned to either a health coaching (HC), or health education (HE) control group. The HC group received coaching to address the lifestyle areas known to be linked to AD risk, such as diet, exercise, sleep, stress, cognitive training, and social interaction. Blood markers of each individual have been assessed to determine carrier status of Apolipoprotein E 4 (APOE4) due to the correlation between the allele and increased risk of Alzheimer’s. To be included in the study, the participants must have at least 2 positive risk factors for AD as determined by the Australian National University-Alzheimer Disease Risk Index (ANU-ADRI). Among non-APOE carriers, a 2x3 (group x time) mixed ANOVA revealed a significant interaction effect wherein the HC group improved DTC score more than the HE group from time 1 to time 3, F (2, 362) =1.253, P=0.017. No significant interaction was noted for APOE carriers.

